# LINC00968 can inhibit the progression of lung adenocarcinoma through the miR-21-5p/SMAD7 signal axis

**DOI:** 10.18632/aging.104011

**Published:** 2020-11-04

**Authors:** Yuxing Zhu, Hao Bo, Zhizhao Chen, Jingjing Li, Dong He, Mengqing Xiao, Liang Xiang, Long Jin, Jianda Zhou, Lian Gong, Yanhong Zhou, Ming Zhou, Wei Xiong, Xiaowei Xing, Ruhong Li, Ke Cao

**Affiliations:** 1 Department of Oncology, Third Xiangya Hospital of Central South University, Changsha 410013, China; 2 Department of Plastic Surgery, Xiangya Hospital, Central South University, Changsha 410008, Hunan, China; 3 Institute of Reproductive and Stem Cell Engineering, Central South University, Changsha 410083, Hunan, China; 4 Department of Respiration, The Second People’s Hospital of Hunan Province of Hunan University of Chinese Medicine, Changsha 410000, China; 5 Department of Plastic Surgery, Third Xiangya Hospital, Central South University, Changsha 410013, Hunan, China; 6 Cancer Research Institute and Key Laboratory of Carcinogenesis of Ministry of Health, Central South University, Changsha 410078, China; 7 Center for Medical Experiments, Third Xiangya Hospital, Central South University, Changsha 410013, China; 8 Yan’an Affiliated Hospital of Kunming Medical University, Kunming 650051, China

**Keywords:** lung adenocarcinoma (LUAD), LINC00968, prognosis, SMAD7, metastasis

## Abstract

Background: Long non-coding RNAs (LncRNAs) have been associated with several types of cancer. However, little is known about their role in lung adenocarcinoma (LUAD).

Results: LINC00968 was significantly differentially expressed in LUAD tissues. Downregulated LINC00968 was associated with clinicopathological features of LUAD. LINC00968 inhibited cell growth and metastasis by regulating the Hippo signaling pathway We demonstrated that LINC00968 acts as a ceRNA to consume miR-21-5p, enhancing the accumulation of SMAD7, a miR-21-5p target.

Conclusions: LINC00968 limits LUAD progression via the miR-21-5p/SMAD7 axis and may serve as a prognostic biomarker and therapeutic target for LUAD.

Methods: We conducted comprehensive data mining on LINC00968 based on the Gene Expression Omnibus (GEO) and The Cancer Genome Atlas (TCGA) database. The expression of LINC00968 in LUAD cells was determined using *in situ* hybridization. We detected LINC00968 function in LUAD cells using the MTT, clone formation, and transwell assays, and tumor xenografts. Label-free quantitative proteomics, western blotting, a dual-luciferase reporter assay, immunofluorescence, and RNA immunoprecipitation assays were used to determine the correlations among LINC00968, miR-21-5p, and SMAD7. Gain- and loss-function approaches were used to explore the effects of LINC00968, miR-21-5p, and SMAD7 on cell proliferation, migration, and invasion.

## INTRODUCTION

Non-small cell lung cancer (NSCLC) is responsible for most cancer-related deaths worldwide, accounting for approximately 80% of all lung cancer cases [1]. Lung adenocarcinoma (LUAD) is now the most frequent subtype of NSCLC, accounting for 50% of the cases [2]. LUAD carries a wide range of genetic and epigenetic alterations, resulting in a major shift in the therapeutic research toward targeted therapies guided by key mutations and targetable molecular drivers. Of note, however, LUAD has a high risk of developing distant metastasis due to the significantly reduced survival time of patients [3]. Hence, the identification of new therapeutic targets, to prevent the disease progression and metastasis of LUAD, is desired.

Long non-coding RNAs (lncRNAs), part of a novel class of non-coding RNAs, are characterized by a transcript length greater than 200 nucleotides in the human transcriptome [4]. LncRNAs have opened a new perspective on the alteration of cellular functions such as cell cycle, apoptosis, and metastasis at the transcriptional level [5]. Recent findings show the occurrence of thousands of changes in the lncRNA expression in various types of cancer, suggesting that their dysregulation may function as potential biomarkers or therapeutic targets for cancer [6]. However, mechanisms that control disease progression remain poorly understood.

Owing to the rapid development of genome-wide sequencing technology, lncRNAs can be quantitatively identified using RNA sequencing (RNA-Seq) and microarray data. However, data have not been extracted effectively from most existing lncRNA expression profiles. Data mining from published microarrays and RNA-Seq data in a systematic way is a highly effective method for identifying key molecules. Based on this, we obtained lncRNA microarray data of LUAD from the online databases, Gene Expression Omnibus (GEO), The Cancer Genome Atlas (TCGA), and the Atlas of Noncoding RNAs in Cancer (TANRIC), to easily access sequence characteristics [7–10]. Here, we validated the significant downregulation of LINC00968 in LUAD tissues among hundreds of dysregulated lncRNAs based on the GEO database. To determine the value of LINC00968 as a clinical biomarker and therapeutic target, we systematically re-analyzed the published microarray and RNA-Seq data and investigated its functions using various cell-based assay in LUAD.

## RESULTS

### The expression levels and clinical value of LINC00968 in LUAD across databases

**Figure 1:**
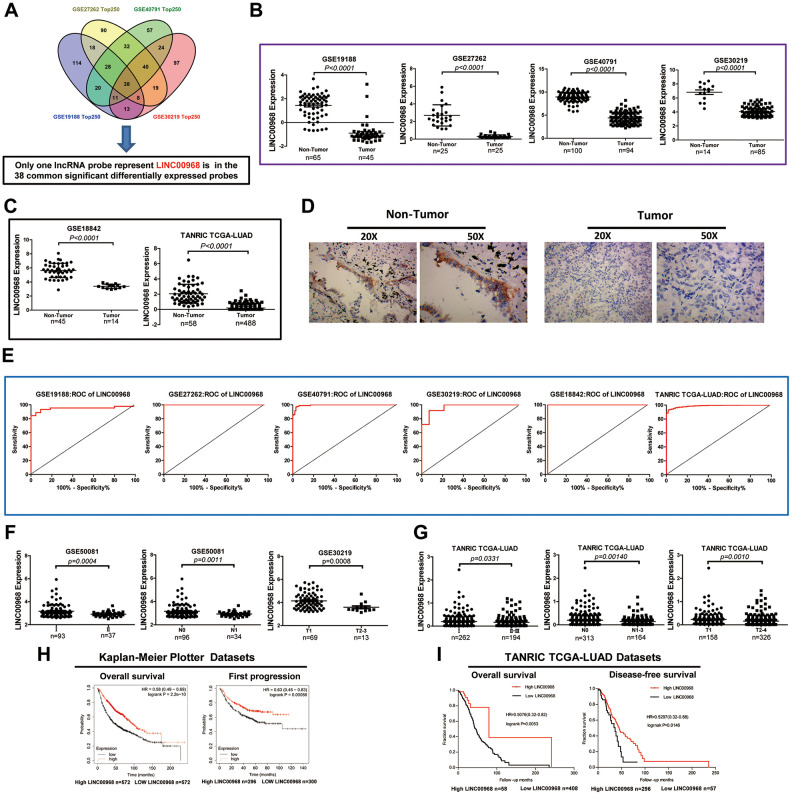
**Decreased LINC00968 is related to the patient's pathological grade and prognosis in LUAD.** (**A**) Identification of common significantly differentially expressed lncRNAs from four independent LUAD microarray analyses using the GEO2R analysis online tools. (**B**) The levels of LINC00968 expression were downregulated in tumor tissues compared with that of non-tumor lung tissues (GSE19188, GSE27262, GSE40791, and GSE30219). (**C**) LINC00968 expression in LAC and non-tumor tissues based on the data from the GSE18842 and TANRIC databases. (**D**) The levels of LINC00968 expression were validated by *in situ* hybridization histochemistry in tissue biopsies. (**E**) The area under the curve (AUC) of LINC00968 levels in LAC patients based on a microarray, ranged from 0.9501 to 1.000, proving that LINC00968 was an effective diagnostic molecular marker for LAC patients. (**F**) Low LINC00968 expression was associated with advanced disease stages in the GSE50081 and GSE30219 datasets (clinical stages, N stages, and T stages). (**G**) Based on the TANRIC database, the relationship between LINC00968 expression and advanced disease stages was further validated (clinical stages, N stages, and T stages). (**H**) Kaplan-Meier overall survival curves and Kaplan-Meier estimates of time to the first progression for patients with LAC classified according to relative LINC00968 expression level. (**I**) Low expression of LINC00968 was associated with shorter overall survival and disease-free survival time.

The GEO2R online analysis tool was used to filter the overlapping lncRNAs among GSE19188, GSE40791, GSE30219, and GSE27262. We found that a total of 38 common significantly differentially expressed RNAs were filtered out by analysis of the four GEO datasets in human LUAD (Figure 1A). Of the 38 genes identified, LINC00968 was the only lncRNA. Subsequently, we assessed five lncRNA expression datasets from GEO and found that LINC00968 was significantly downregulated in LUAD tumor tissues (T) compared to the non-tumor tissues (N) (GSE19188 N n=65 vs. T n=45, GSE27262 N n=25 vs. T n=25), GSE40791 N n=100 vs. T n=94), GSE30219 N n=14 vs. T n=85, GSE18842 N n=45 vs. T n=14; Figure 1B). Consistent with the above data, the results from the TANRIC databases (Figure 1C) indicated a significant decrease in LINC00968 expression in LUAD tissues (n=488) compared with the non-tumor lung tissues (n=58). We further confirmed that LINC00968 was downregulated in human LUAD tissues using *in situ* hybridization (Figure 1D). Receiver operating characteristic (ROC) curve analysis indicated that the areas under the curve (AUC) were 0.9501, 1, 0.9944, 0.9681, 0.9778, and 0.9877 from GSE19188, GSE27262, GSE40791, GSE30219, GSE18842, and TANRIC, respectively, for LINC00968 (P<0.0001; Figure 1E).

We further analyzed the relative expression of LINC00968 in patients with LUAD at different disease stages from GEO (GSE50081 and GSE30219). As shown in Figure 1F, LINC00968 expression was remarkably lower in LUAD tissues obtained from patients with advanced clinical stages (II, n=37), advanced N stages (N1, n=34), and advanced T stages (T2-3, n=13), compared with that of individuals at earlier clinical (I, n=93), advanced N (N0, n=96), and advanced T (T1, n=69) stages, respectively. Consistently, lower LINC00968 expression was associated with advanced clinical, T, and N stages, in data retrieved from the TANRIC database (Figure 1G). Low LINC00968 expression was associated with poor pathologic differentiation, disease relapse, and recurrence rates (Supplementary Figure 1). We further detected the prognostic value of LINC00968 in LUAD based on the Kaplan-Meier Plotter and TANRIC databases. The Kaplan-Meier survival analysis of Kaplan-Meier Plotter datasets showed that overall survival (OS) and first progression times of patients with low LINC00968 expression were significantly shorter than that of patients with high LINC00968 expression (Figure 1H). The survival curves for OS and disease-free survival (DFS) times are presented in Figure 1I, demonstrating that low LINC00968 expression was associated with shorter survival time.

### Upregulation of LINC00968 inhibits cell proliferation, migration, and invasion

**Figure 2:**
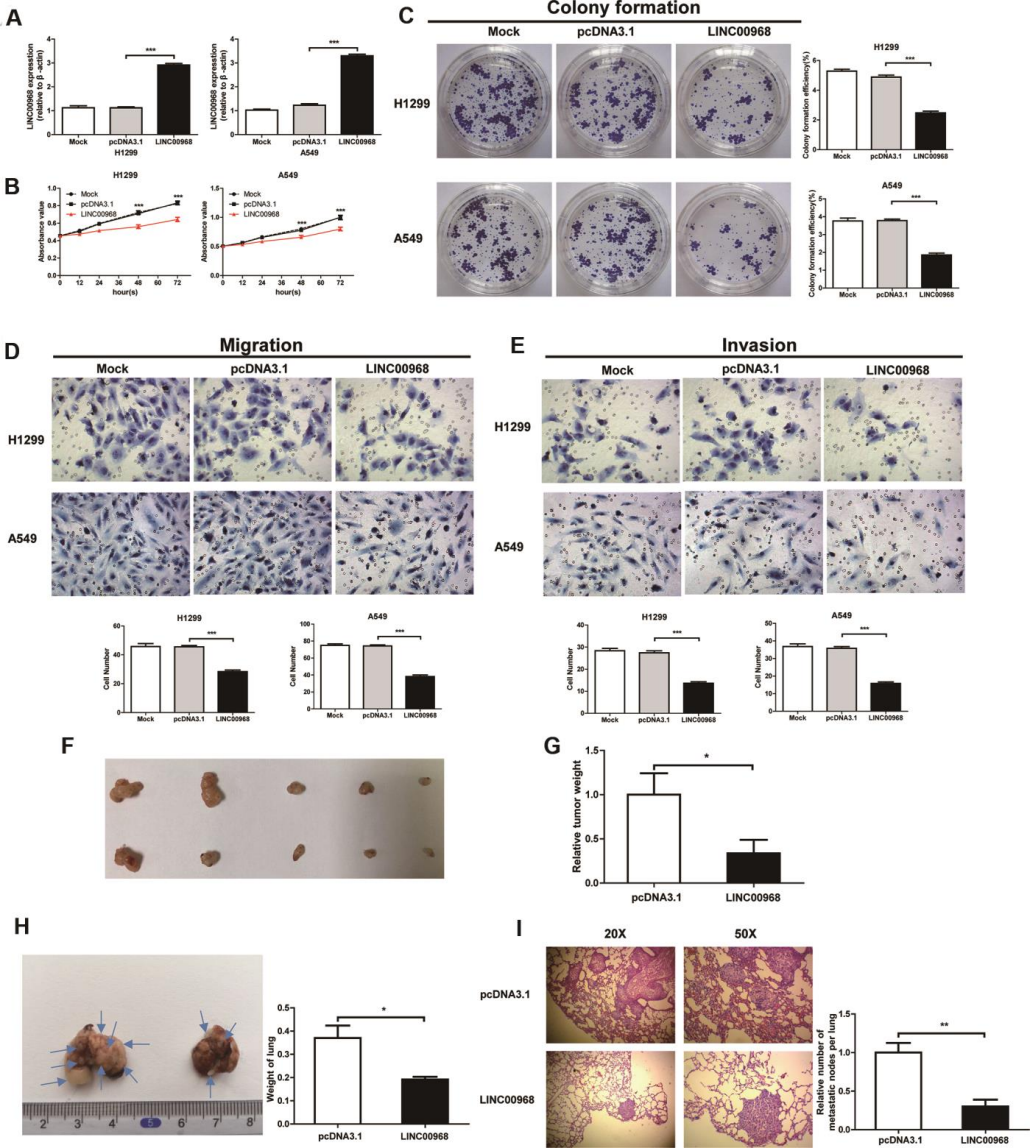
**LINC00968 inhibits malignant phenotype in tumor cells.** (**A**) After treatment with LINC00968 overexpressing or control plasmids, the LINC00968 expression levels were validated by qTR-PCR. (**B**) Cell proliferation was detected at the indicated time points. (**C**) Representative images of cell forming colonies and colony formation efficiency of each group. (**D**) The migration ability of LINC00968 overexpressing cells in each group. The histogram of the migration cell number is on the right. (**E**) The representative images of cells moving across the Matrigel membranes. (**F**) Tumor xenografts from nude mice. (**G**) Upregulation of LINC00968 significantly decreased tumor weight. (**H**) Representative images of the lung metastatic foci on the surface area. Arrows indicate the lung metastatic foci (left). The lung weights in each group are listed to the right. (**I**) Representative images of lung metastatic foci after HE-staining (left). The number of metastatic foci (right). *P<0.05, **P<0.01, ***P<0.001.

We detected the levels of LINC00968 in both H1299 and A549 cells treated with LINC00968 overexpressing plasmids and compared them with cells either untreated or treated with empty plasmids. overexpressing H1299 and A549 cells treated with LINC00968 overexpressing plasmids had an approximate 3-fold increase in levels of LINC00968 (Figure 2A). LUAD cell proliferation was significantly inhibited by LINC00968 overexpression (Figure 2B). As expected, colony formation was reduced by the upregulation of LINC00968 (Figure 2C). We then investigated the effects of the upregulation of LINC00968 on migration and invasion. Cells exposed to LINC00968 overexpressing plasmids exhibited an obvious decrease in cell migration (Figure 2D) and invasion (Figure 2E) in both H1299 and A549 cells. LINC00968 knockdown treatment exerted a slight increase in cell proliferation, migration, and invasion in LUAD cells. However, no effects were observed on cell colony formation (Supplementary Figure 2).

### Upregulation of LINC00968 inhibits cell growth and metastasis *in vivo*

Then, LUAD cells were implanted into nude mice by subcutaneous injection to detect the effect of LINC00968 on tumor growth. The results showed that the treatment with LINC00968-overexpressing plasmids inhibited the weight of xenograft tumors (pcDNA3.1: 1±0.2436, n=5; LINC00968: 0.3367±0.1526, n=5) in A549 cells (Figure 2F, 2G). To evaluate the functions of LINC00968 during metastasis of LAC cells, LINC00968-overexpressing A549 cells were delivered intravenously. After 50 days of injection, we found that the overexpression of LINC00968 led to the downsizing of the metastatic foci on the lung surface as well as lung weight (Figure 2H) as compared to control groups (pcDNA3.1: 0.37±0.05413, n=5; LINC00968: 0.1920±0.01114, n=5). H&E staining slices further confirmed that the upregulation of LINC00968 significantly diminished the size of lung metastasis nodules (Figure 2I). Lung nodules were decreased in LINC00968-overexpressing cells compared with the control group (Figure 2I, pcDNA3.1: 1±0.1252, n=5; LINC00968: 0.3023±0.08702, n=5). These findings in lung metastasis models indicated that the upregulation of LINC00968 significantly suppressed the tumor growth and metastasis.

### LINC00968 enhances SMAD7 expression in LUAD

**Figure 3:**
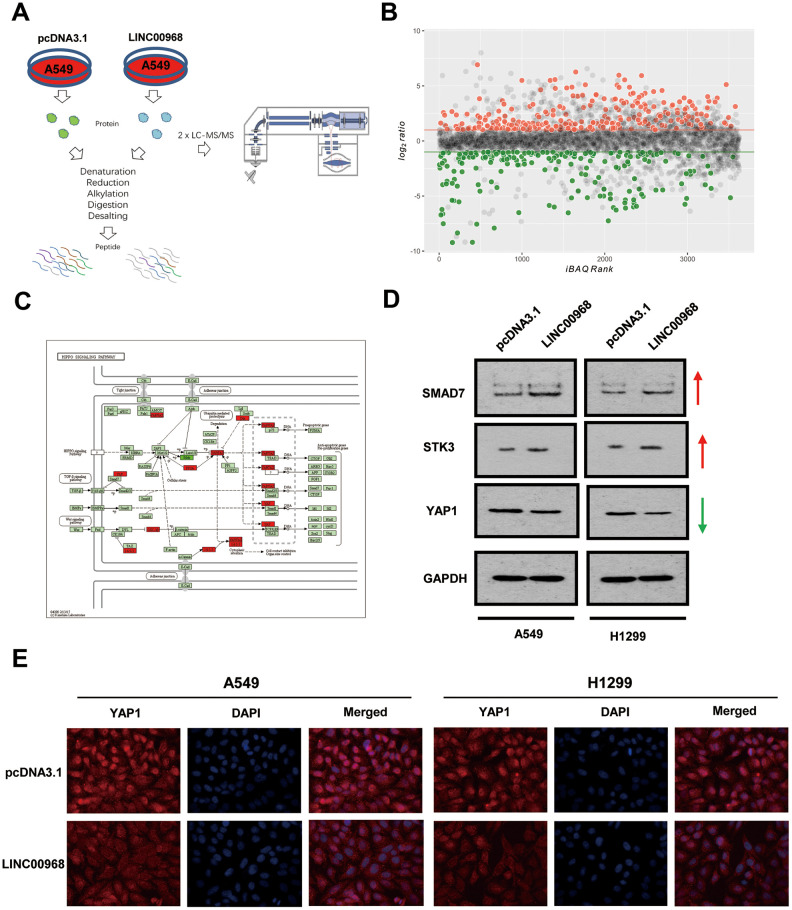
**LINC00968 regulates the SMAD-mediated Hippo signaling pathway.** (**A**) The workflow of random peptide arrays used to screen downstream proteins specifically regulated by LINC00968 in LUAD. (**B**) Volcano plots of the differentially expressed lncRNAs (266 upregulated and 208 down-regulated proteins) in LINC00968 overexpressing and control A549 cells. (**C**) The most frequent proteins and relevant signaling pathways regulated by LINC00968. (**D**) SMAD and STK3 expressions were increased whereas YAP1 was decreased after LINC00968 upregulation as detected by western blotting. (**E**) YAP1 expression (red), validated by immunofluorescence following LINC00968 overexpression in LUAD cells.

Label-free quantitative proteomic analysis was used to screen the downstream peptide specifically regulated by LINC00968 in LUAD. The workflow diagram is shown in Figure 3A. We identified 266 upregulated and 208 downregulated proteins in A549 cells (Figure 3B). The most frequent proteins and relevant signaling pathways are summarized in Figure 3C using the KEGG software (https://www.kegg.jp/kegg/pathway.html). Western blotting further confirmed that SMAD7 and STK3 were increased whereas YAP1 was reduced following treatment with LINC00968 overexpressing plasmids (Figure 3D), suggesting that LINC00968 enhances the inhibitory action of Smad7 on the Hippo signaling pathway. Immunofluorescence detected lower YAP1 expression in LINC00968 overexpressing A549 and H1299 cells compared with that of the control cells (Figure 3E), suggesting that LINC00968 upregulation may inhibit YAP1 expression.

### LINC00968 enhances SMAD7 expression by sponging miR-21-5p

**Figure 4:**
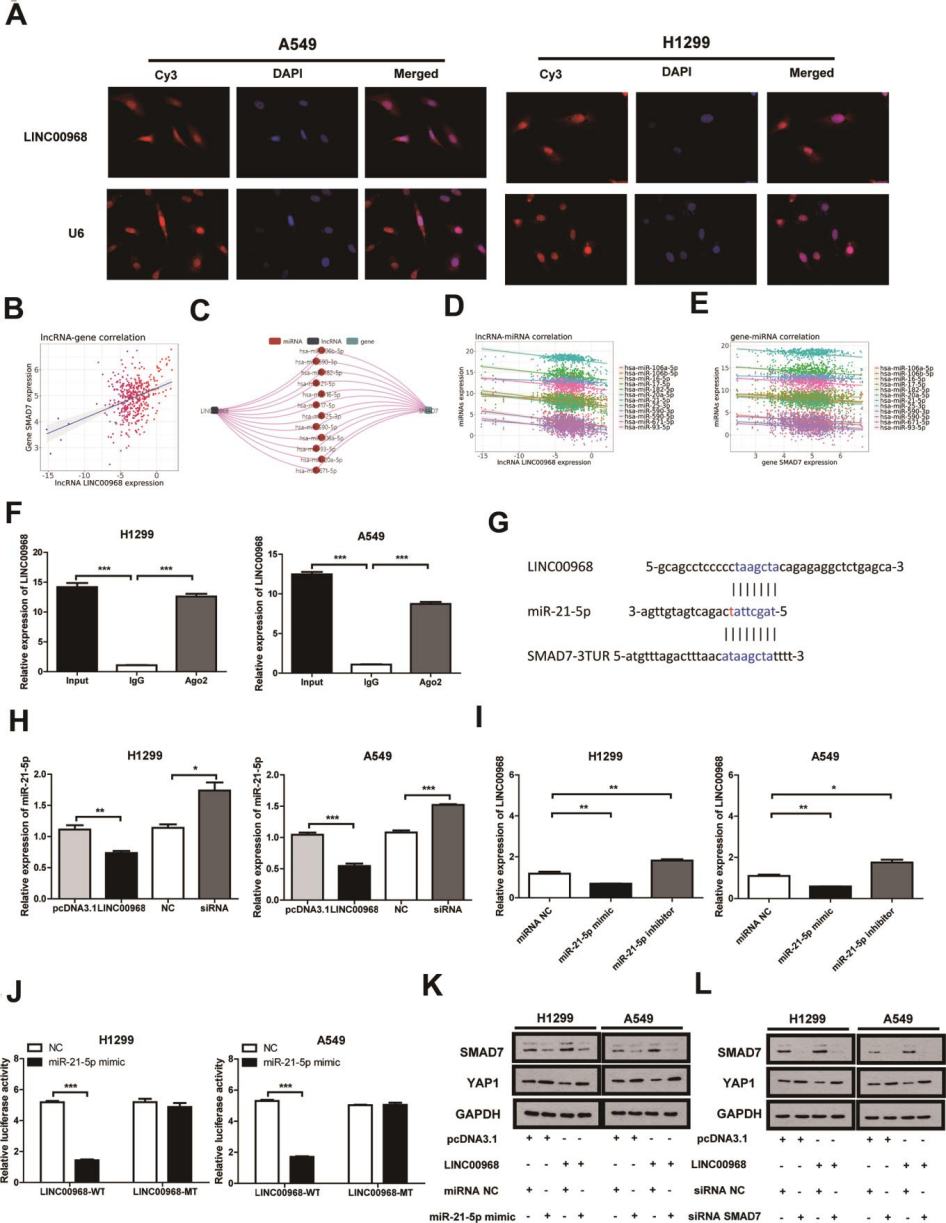
**LINC00968 inhibits tumor development by regulating the miR-21-5p/SMAD7 axis.** (**A**) FISH detection of subcellular localization of LINC00968 in A549 and H1299 cells. (**B**) The miRNACancerMAP datasets suggested that there was a significant positive correlation between LINC00968 and SMAD7. (**C**) The miRNAs negatively related to LINC00968 and SMAD7 most frequently were summarized by miRNACancerMAP. (**D**) Correlation between LINC00968 and the top 12 most frequent miRNAs. (**E**) Correlation between SMAD7 and the top 12 most frequent miRNAs. (**F**) qRT-PCR quantification of LINC00968. LINC00968 was more enriched in the cells treated with the Ago2 antibody compared with those treated with the IgG negative control antibody. (**G**) The complementary sites of LINC00968, miR-21-5p, and SMAD7. (**H**) The expression of miR-21-5p was decreased and increased in LINC00968 overexpressing and knockdown LUAD cells, respectively. (**I**) LINC00968 expression was decreased in the miR-21-5p mimics group and increased in the miR-21-5p inhibitor group. (**J**) H1299 and A549 cells co-transfected with a miR-21-5p mimic or negative control (NC) and luciferase vectors containing either wild type (LINC00968-WT) or mutated (LINC00968-mut) miR-21-5p-binding sites. The luciferase activity was measured after 48 h. (**K**) and (**L**) SMAD7 and YAP1 protein expression following LINC00968, SMAD7 and/or miR-21-5p modulation in A549 and H1299 cells. *P<0.05, **P<0.01, ***P<0.001.

RNA FISH indicated that LINC00968 localizes to the cytoplasm and nucleus of both A549 and H1299 cells (Figure 4A). Data from the miRNACancerMAP online database (http://cis.hku.hk/miRNACancerMAP/) suggested that there was a significant positive correlation between LINC00968 and SMAD7 (Figure 4B). It is well known that lncRNAs often compete for endogenous RNA (ceRNA) and target miRNAs. Here, we investigated the lncRNA-miRNA-mRNA network in relation to LINC00968 and SMAD7. We used miRNACancerMAP to filter the most frequent miRNAs, of which, the top 12 are illustrated in Figure 4C. We further analyzed the most frequent miRNAs negatively associated with LINC00968, of which the top 12 are illustrated in Figure 4D. We analyzed the miRNAs negatively associated with SMAD7 most frequently (Figure 4E). The candidate miRNA identified, miR-21-5p is well-known to function as a tumor promoter and directly targets SMAD7 in lung cancer [11, 12]. Thus, we focused on whether LINC00968 binds to miR-21-5p, exerting a ceRNA effect. RIP assays indicated that a significant increase in LINC00968 was observed in both A549 and H1299 cells treated with the Ago2 antibody compared with that of those treated with IgG negative control antibody (Figure 4F). The complementary sites among LINC00968, miR-21-5p, and SMAD7 were recognized using miRcode (https://omictools.com/mircode-tool) and are presented in Figure 4G. The miR-21-5p mimics, inhibitors, and SMAD7 siRNAs were transfected into LUAD cells (Supplementary Figure 3). Subsequently, miR-21-5p expression was reduced in LINC00968 overexpressing LUAD cells, whereas it increased in LINC00968 knockdown cells (Figure 4H). As expected LINC00968 expression was decreased in the miR-21-5p mimics group whereas it was enhanced in the miR-21-5p inhibitor group (Figure 4I). To confirm the combination between LINC00968 and miR-21-5p, luciferase assays were performed. As expected, the luciferase activity of the LINC00968-WT vector was significantly reduced by the addition of miR-21-5p mimics compared with that of the NC cells. However, miR-21-5p-mediated repression of luciferase activity was not found when cells were transfected with the LINC00968-MT vector (Figure 4J). SMAD7 and YAP1 expression was restored in A549 and H1299 cells transfected with a LINC00968 overexpression vector by co-transfection with miR-21-5p mimics (Figure 4K). Similarly, SMAD7 and YAP1 expression was restored in A549 and H1299 cells transfected with a LINC00968 overexpression vector by co-transfection with SMAD7 siRNA (Figure 4L). These results confirmed that LINC00968 enhanced SMAD7 expression by sponging miR-21-5p.

**Figure 5:**
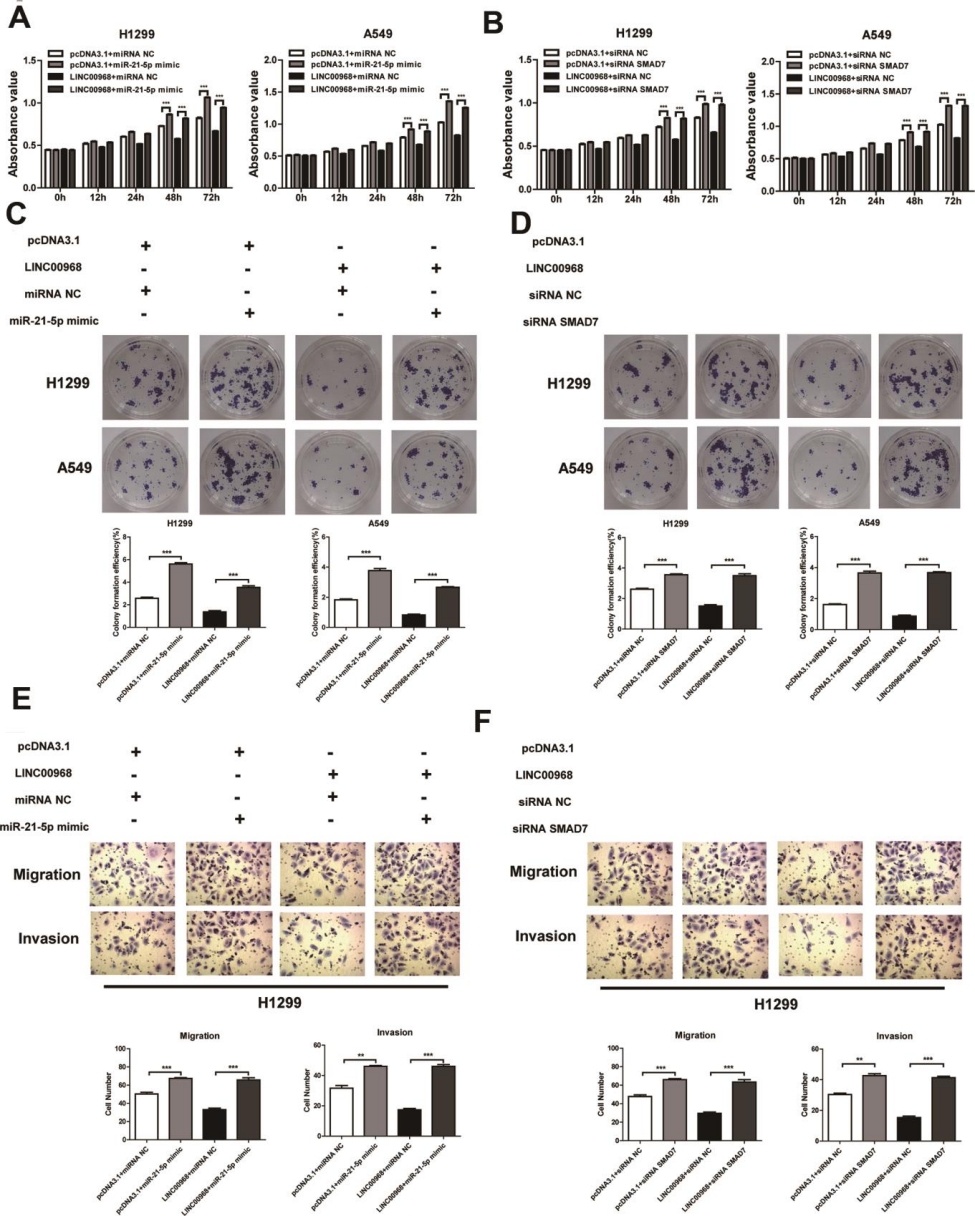
**LINC00968 inhibits tumor development via sponging miR-21-5p/SMAD7 axis.** The miR-21-5p mimics significantly increased cell proliferation (**A**), colony-forming ability (**C**), invasion, and migration (**E**), and also partially attenuated the inhibitory effects of LINC00968 over-expression. SMAD7 knockdown enhanced cell proliferation (**B**), colony-forming ability (**D**), invasion, and migration (**F**), and also partially attenuated the inhibitory effects of LINC00968 over-expression. **P<0.01, ***P<0.001.

To investigate the effect of the LINC00968/miR-21-5p axis on the malignant phenotype of LUAD, LINC00968 overexpressing plasmids and miR-21-5p mimics were co-transfected into tumor cells. Results of *in vitro* experiments showed that the upregulation of miR-21-5p significantly increased cell proliferation (Figure 5A), colony-forming ability (Figure 5C), invasion, and migration (Figure 5E and Supplementary Figure 4A), whereas upregulation of miR-21-5p partially attenuated the inhibitory effects of LINC00968 overexpression. No differences were observed in both the empty pcDNA3.1 plasmid and the control miRNA groups. Additionally, SMAD7 knockdown partially debilitated the anti-proliferation (Figure 5B), anti-forming ability (Figure 5D), anti-migration, and anti-invasion (Figure 5F and Supplementary Figure 4B) effects of LINC00968 overexpression. Taken together, LINC00968 promoted SMAD7 expression through direct interaction with miR-21-5p.

### Expression level and clinical value of miR-21-5p and SMAD7 in LUAD

**Figure 6:**
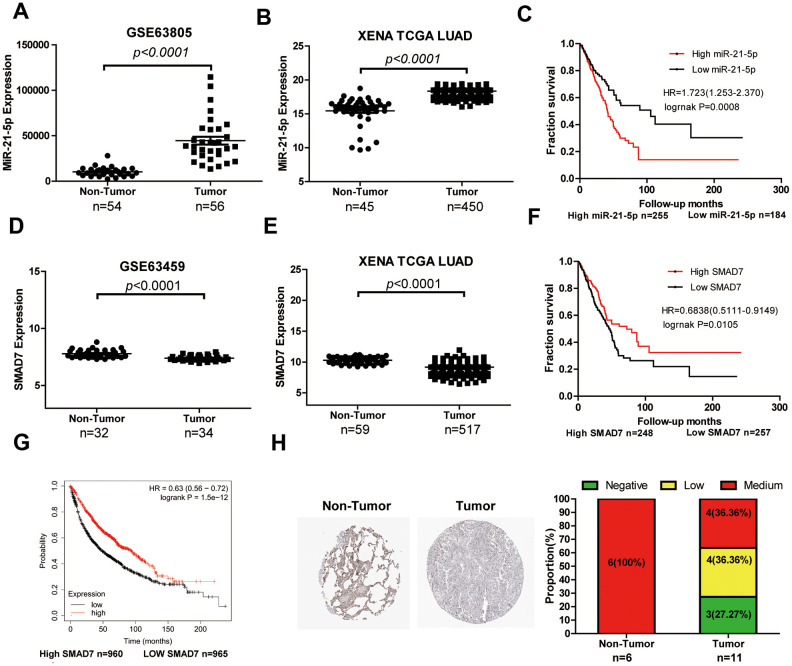
**The expression level and clinical value of miR-21-5p and SMAD7 in LUAD.** (**A**) The miR-21-5p expression was significantly increased in LUAD tissues compared with that of non-tumor tissues from the LUAD chip GSE63805 dataset (**A**) and XENA database (**B**). (**C**) The survival curve analyses indicated that high expression of miR-21-5p was associated with poor clinical outcomes. SMAD7 expression decreased in tumor tissues compared with non-tumor lung tissues from the GSE63459 dataset (**D**) and XENA database (**E**). The Kaplan Meier-plotter (**F**) and XENA (**G**) database demonstrated that the low SMAD7 expression was associated with significantly poor patient survival. (**H**) SMAD7 protein expression was downregulated in human LUAD tissue biopsies as evidenced by immunohistochemical staining from the Human Protein Atlas Database.

LUAD tissue chip GSE63805 (Figure 6A) and XENA databases (Figure 6B) were used to estimate the differential expression of miR-21-5p. The miR-21-5p expression was significantly increased in LUAD tissues compared with that of the non-tumor tissues. The survival curve analysis indicated that high expression of miR-21-5p was closely related to the poor prognosis in patients with LUAD (Figure 6C). Subsequently, SMAD7, the target of miR-21-5p, was expressed poorly in tumor tissues (n=33) compared with that of non-tumor lung tissues (n=32) (Figure 6D). We comprehensively analyzed SMAD7 expression using the XENA database (Figure 6E) and found that SMAD7 was downregulated in tumor tissues (n=517) compared with non-tumor lung tissue (n=59). The XENA (Figure 6F) and Kaplan-Meier Plotter (Figure 6G) database showed that low SMAD7 expression was associated with significantly poor patient survival. We further confirmed that the SMAD7 protein was indeed downregulated in human LUAD tissues as evidenced by the Human Protein Atlas Database (https://www.proteinatlas.org/) (Figure 6H).

## DISCUSSION

Here, we identified differential expression levels of LINC00968 in LUAD using comprehensive data mining of various databases, such as GEO, TCGA, and TANRIC. We identified molecules that are generally considered critical, universality for disease progression [13]. We found that LINC00968 was significantly downregulated in LUAD tissues, as evidenced by large-scale tissue samples. Two recent studies reported that LINC00968 is among the top 10 aberrantly expressed lncRNAs and has decreased expression in non-small cell lung cancer [14, 15]. However, little is known about the clinical relevance and molecular involvement of LINC00968 in LUAD progression. The AUC values of the ROC curve ranged from 0.9501 to 1.000, proving that LINC00968 was an effective diagnostic molecular marker in patients with LUAD. Low levels of LINC00968 expression are associated with poor clinical outcomes, as LINC00968 has decreased expression in the advanced tumor TNM stage, tumor cell differentiation, neoplasm recurrence, and poor patient survival. These findings strongly imply that the deletion of LINC00968 contributes to LUAD tumor development and progression. However, the detailed mechanisms remain unclear.

We used A549 and H1299 cells to detect the *in vitro* and *in vivo* effects of LINC00968 on tumor growth and metastasis. We demonstrated that the upregulation of this lncRNA significantly inhibited cell proliferation, migration, and invasion. We also noted reduced xenograft weight and identified fewer metastatic foci in tumor-bearing animals. These results were consistent with the report by Xiu DH [16] in breast cancer. Interestingly, only slight facilitation of proliferation, migration, and invasion was observed in tumor cells after LINC00968 knockdown. We speculate that LINC00968 knockdown may not cause significant changes in the malignant phenotype based on the intrinsic endogenous expression of LINC00968 is quite low, as confirmed by lncRNA microarray data [17].

Label-free quantitative proteomics was used to screen the downstream proteins specifically regulated by LINC00968 in LUAD. We identified several key molecules that were subsequently changed after overexpression of LINC00968, involving the Hippo signaling pathway, such as SMAD7, STK3, and YAP1. The Hippo signaling pathway is mainly a SMAD-dependent pathway that is widely involved in several cellular processes, including cell growth and cell differentiation [18]. SMAD7 is accepted as a product required for negative feedback in Hippo signaling [19]. YAP1 expression can be both negatively and positively correlated with Smad7 even within the same cell types, depending on the disease stage [20]. The reason for this is unknown. We found that the upregulation of LINC00968 inhibited the expression of YAP1, confirmed by immunofluorescence. The positive relationship between LINC00968 and Smad7 demonstrated suggests an inverse relationship between YAP1 and Smad7. Here, we first demonstrated that the upregulation of LINC00968 inhibited the SMAD7-mediated Hippo signaling pathway.

Since 2007, the first ceRNA hypothesis was reported that the non-protein coding gene IPS1 RNA increased PHO2 mRNA through complementary binding to miR-399 [21]. This garnered great interest globally in deciphering the mechanism of action of lncRNAs [22]. Experimental evidence supporting the lncRNA-miRNA-mRNA network provides a new visual angle for understanding the tumor cell process. With the help of large-scale bioinformatics data mining, we found that miR-21-5p had a strong negative relation to both LINC00968 and SMAD7 expression, as confirmed by qRT-PCR and RIP assays. miR-21-5p has been reported to have pro-proliferation, pro-metastatic, and anti-apoptotic effects in LUAD [12, 22]. On the other hand, miR-21-5p directly binds to SMAD7 mRNA, as evidenced by the luciferase assay in non-small cell lung cancer [11]. Although upregulation of miR-21-5p significantly promoted cell proliferation, invasion, and migration, it also partially attenuated the inhibitory effects of LINC00968 overexpression. Consistent with the above previous findings, miR-21-5p was highly expressed whereas SMAD7 expression decreased in LUAD tissues. The increase and decrease in levels of miR-21-5p and low SMAD7 were associated with poor clinical outcomes, confirmed by large-scale data mining of the GEO and XENA databases. These findings indicated that LINC00968 may serve as a ceRNA for miR-21-5p, enabling the suppression of Hippo signaling.

In conclusion, we demonstrated that LINC00968 acts as a ceRNA to consume miR-21-5p, leading to enhanced accumulation of SMAD7. The identified LINC00968 may serve as a valuable diagnostic and prognostic biomarker as well as a therapeutic target for patients with LUAD.

## MATERIALS AND METHODS

### Data mining based on the GEO, TANRIC, and XENA databases

The lncRNA expression profiles in human LUAD tissues based on microarray and RNA-seq data were collected from GEO, TCGA, and TANRIC databases. Each dataset was manually curated and filtered to identify the key lncRNAs. We used a total of eight (GSE19188, GSE27262, GSE40791, GSE30219, GSE18842, GSE50081, GSE63805, and GSE63459) and five (GSE19188, GSE27262, GSE40791, GSE30219, and GSE18842) datasets from LUAD microarrays and LUAD receiver operating characteristic (ROC) curves, respectively. We used the Kaplan Meier-plotter online tool to analyze the survival curves of differentially LINC00968-expressing patients with LUAD. The data were obtained from GEO and integrated by Kaplan–Meier plotter [23]. TANRIC and XENA are open-access tools for interactive exploration of lncRNAs based on TCGA RNA-seq data and the clinical information associated with LUAD samples such as the patient’s survival time, TNM stage, and clinical stage. TANRIC data were analyzed according to a previously described method [10]. The lncRNA–gene and lncRNA-miRNA correlations were filtered using the miRNACancerMAP (http://cis.hku.hk/miRNACancerMAP/) platform as previously described [24]. Immunohistochemical (IHC) images of human LUAD and non-tumor tissues were obtained from an online tissue-based map database [25] (The Human Protein Atlas, https://www.proteinatlas.org/).

### Human tissue samples, cell lines, and transfection

LUAD (n=30) and normal, non-tumor tissue specimens (n=30) were obtained from patients who underwent their first lung cancer resection at the Third Xiangya Hospital, China. All specimens were subjected to *in situ* hybridization for LINC00968 detection. This study was approved by the Ethics Committee of the Third Xiangya Hospital, Central South University (Changsha, China). We purchased the human non-small cell lung carcinoma cell lines H1299 and A549 from ATCC (Manassas, VA, USA). These two LUAD cell lines were cultured in DMEM supplemented with 10% FBS (Gibco; Waltham, MA, USA). The sequence of LINC00968 was cloned into pcDNA3.1. The recombinant plasmids were transfected into H1299 and A549 cells using Lipofectamine 3000 (Invitrogen, Carlsbad, CA). Cells transfected with empty vectors were used as controls. The miR-21-5p mimics and inhibitors were purchased from Ribobio (Guangzhou, China) and transfected into LUAD cells using Lipofectamine 3000. After 48 h, qRT-PCR was used to detect the intracellular expression of LINC00968.

### Quantitative real-time polymerase chain reaction (qRT-PCR)

Total RNA was extracted from cells using a TRIzol reagent (Thermo Fisher Scientific, Waltham, USA). RNA was reverse transcribed into cDNA using the iScript cDNA Synthesis Kit (Bio-Rad, Hercules, USA). The following primers were used: LINC00968 F: 5′-GCCCAGTTGACAGGAAATGT-3′ and R 5′-TTGGTTCTCAATGGGATGGT-3′. GAPDH F: 5′- GGAGCGAGATCCCTCCAAAAT-3′ and R 5′- GGCTGTTGTCATACTTCTCATGG-3′. miR-21-5p F: 5′- ACACTCCAGCTGGGTAGCTTATCAGACTGA-3′ and R 5′- CTCAACTGGTGTCGTGGAGTCGGCAATTCAGTTGAGTCAACATC-3′. U6snRNA F: 5′-CTCGCTTCGGCAGCACA-3′ and R 5′- AACGCTTCACGAATTTGCGT-3′. The expression of LINC00968 and miR-21-5p were normalized using β-actin and U6 as references, respectively. The relative expression levels were calculated by the ^−ΔΔ^Ct method.

### MTT cell proliferation assay

Cells were seeded at a density of 1×10^4^ cells per well in 96-well plates. MTT (Beyotime, Shanghai, China) solution (10 μL) was added to each well at 0, 12, 24, 36, 48, and 96 h, respectively. The cells were incubated at 37 °C for 4 h. Then, this MTT solution was removed and 100 μL of formazan solution was added and incubated at 37 °C for another 4 h. Cell proliferation was determined using a microplate reader at a wavelength of 570 nm.

### Clone formation assay

Approximately 1000 cells were seeded into 60 mm culture dishes incubated with medium containing 10% FBS and cultured in a humidified atmosphere with 5% CO_2_ at 37 °C. After 3 weeks, the visible colonies were fixed and stained. Visual estimates were used to count the number of colonies in each dish. colony formation efficiency (%) =number of clones formed/number of planted cells×100%

### Transwell migration and invasion assays

The transwell migration assay was used to study the effect of LINC00968 on cell migration, whereas the transwell invasion assay was used to investigate the effect of LINC00968 on cell invasion. LUAD cells (5 × 10^4^) were seeded into upper transwell chambers with 8-μm pores (Corning Inc., Corning, USA) supplemented with DMEM containing 1% FBS, while the lower chambers were supplemented with DMEM containing 15% FBS. For the invasion assay, the upper transwell chambers were coated with Matrigel matrix. After incubation of cells for 48 h, the cells were fixed and stained. The cell numbers in the lower chambers were counted.

### Tumor xenograft

Animal work was carried out at Hunan SJA Laboratory Animal Co., Ltd. All mice (n=20) were purchased from this company. Mice were housed under SPF conditions for 3-5 days. Male BALB/c nude mice weighing 18–22 g were subjected to tumor implantation. A549 cells (5×10^6^/mouse) were washed and suspended in 200 μL of serum-free medium. Sodium pentobarbital at a dose of 40 mg kg −1 was used to stupefy mice. When mice lost response, A549 cells were injected into mice (control group: n=5, LINC00968 overexpression group: n=5) subcutaneously. Nude mice were euthanized by injection of excessive sodium pentobarbital on day 30 and tumors were harvested. For lung metastasis models, 2×10^6^ A549 cells were injected via the tail vein after mice (control group: n=5, LINC00968 overexpression group: n=5) were anesthetized. On day 50, after nude mice were euthanized, the lungs were resected and processed for routine H&E staining. Lung tissue samples were fixed with formalin prior to embedding. Hematoxylin and eosin were used to stain the samples and then analyzed by light microscopy. Lung metastatic nodules in each mouse were calculated. All animal experiments were performed per relevant guidelines and regulations and were approved by the Ethics Committee of the Third Xiangya Hospital, Central South University (Changsha, China).

### H&E staining

Briefly, paraffin sections were deparaffinized with xylene, absolute ethanol and alcohol and washed with distilled water. Then put the slices in hematoxylin for 3-8 minutes, rinse with distilled water and differentiate with 1% hydrochloric acid alcohol for a few seconds and use 6% ammonia to turn the slices blue. Next, put the sections in eosin for staining for 1-3 minutes. Finally, the slices were put into 95% alcohol, absolute alcohol and xylene for dehydration. After dehydration, use neutral gum to seal the slices, and observe and analyze the slices with a microscope.

### Dual-luciferase reporter assay

Briefly, the sequences (2518 nt) of LINC00968-wild-type or LINC00968-mutated-type were ligated into vectors pmirGLO (Promega). We used hsa-miR-21-5p mimics (Sense: 5′-UAGCUUAUCAGACUGAUGUUGA-3′, antisense: 5′-AACAUCAGUCUGAUAAGCUAUU-3′) and negative control mimics (Sense: 5′-UUCUCCGAACGUGUCACGUTT-3′, antisense: 5′-ACGUGACACGUUCGGAGAATT-3′). The hsa-miR-21-5p mimics were transfected into H1299 cells with a LINC00968-wild-type vector or a LINC00968-mutated-type vector using Lipofectamine™ 2000. The Renilla luciferase plasmid (pRL-TK, Promega) was used for the normalization of transfection. The luminescence of firefly and Renilla luciferase was detected using the Dual-Luciferase® Reporter Assay System (Promega) following the user manual.

### *In situ* hybridization and fluorescence *in situ* hybridization

LINC00968 location and expression status were evaluated using in situ hybridization (ISH) in tissues of human LUAD tissues. The LINC00968 ISH Probe (3′-GTACTATCTGCTCCATAGAAACCT-5′) was purchased from Ribobio (Guangzhou, China). The probe working solution was incubated with tissues overnight at 37 °C. The positive signal appeared as brown after the color reaction. Fluorescence in situ hybridization (FISH) was performed to detect LINC00968 location and expression in LUAD cells. The red FISH Probe (Ribobio, Guangzhou, China) and blue DAPI for nuclear staining were purchased from Ribobio (Guangzhou, China). Briefly, cells were seeded in 24-well plates (6×10^4^/well) and then fixed with 4% paraformaldehyde. Hybridization solution (100 μL) was added to each well, and the reaction time was over 6 h at 37 °C. The signals of each sample were captured by fluorescence microscopy.

### Label-free quantitative proteomics

Briefly, total proteins were extracted from overexpressing LINC00968 A549 cells or control A549 cells. Peptide samples (2 μg) were separated using an EASY-nLC1200 system and then analyzed using a Q Exactive mass spectrometer (120 min/sample), which was performed at Kangchen Biotech (Shanghai, China). Identification of the differentially expressed peptides was set at P < 0.05, fold-change > 2, following gene ontology (GO) enrichment analysis using Blast2Go 4.0.7 software and KEGG pathway analysis by KEGG software.

### Western blotting

Lysis buffer was used to isolate the total proteins that were quantified using a BCA kit (P0011, Beyotime, Shanghai, China). A 10% SDS-PAGE separation gel was used to separate the protein (30 μg/lane) and then transferred onto a PVDF membrane. The PVDF membrane was incubated with primary antibodies followed by incubation with secondary antibodies. The following primary antibodies were used: anti-SMAD7 (Ptgcn, 66478-1-Ig), anti-STK3 (Proteintech, 12097-1-AP), anti-YAP1 (Abcam, ab217693), and anti-GAPDH (Abcam, ab125247).

### RNA immunoprecipitation (RIP)

The anti-AGO RIP was performed using a RIP kit (Thermo Fisher Scientific, MA, USA). Briefly, the cell supernatants were incubated with protein A/G magnetic beads conjugated with anti-SNRNP70 antibody, normal rabbit IgG (Cat. #PP64B) or anti-AGO2 antibody (ab32381) at room temperature for 30 min. Anti-SNRNP70 antibody was used as a positive control, while normal rabbit IgG was used as a negative control. The RNA-binding Protein-RNA complexes (RIP) were incubated at 4 °C overnight. Subsequently, purified RNA was amplified by qRT-PCR to quantify LINC00968. Primers were specific to U1 snRNA. The primers used were as follows: U1: F: 5′-GGGAGATACCATGATCACGAAGGT-3′, R: 5′-CCACAAATTATGCAGTCGAGTTTCCC-3′, LINC00968 F: 5′-GCCCAGTTGACAGGAAATGT-3′, R: 5′-TTGGTTCTCAATGGGATGGT-3′, Homo-GAPDH: F: 5′-GGAGCGAGATCCCTCCAAAAT-3′, R: 5′-GGCTGTTGTCATACTTCTCATGG-3′.

### Immunofluorescence

Immunofluorescence was performed to detect YAP1 location and expression in A549 and H1299 cells. Cells were seeded into 6-well plates (1×10^6^/well). Cells were fixed with 4% paraformaldehyde when the confluence reached 80%. The anti-YAP1 antibody (red) and blue DAPI for nuclear staining were incubated with LUAD cells in the dark. The red signals of each well were captured by fluorescence microscopy.

### Statistical analysis

Data were processed using GraphPad Prism Software (GraphPad Software, San Diego, CA, USA). We used Student’s *t*-tests to determine differences between two groups. We used ANOVA followed by Dunnett’s tests for differences among multiple groups. Statistical significance was set at p-value<0.05.

### Availability of data and materials

The data used or analyzed in this study are available upon reasonable request.

### Ethics approval and consent to participate

The study strictly adhered to the ethical guidelines of the Declaration of Helsinki and was approved by the Ethics Committee of the Third Xiangya Hospital of Central South University. All animal experiments were performed per relevant guidelines and regulations. Informed consent was obtained from all participants included in this study, according to the committee regulations.

### Consent for publication

All authors agree with the contents of the manuscript.

## Supplementary Material

Supplementary Figures

## References

[1] Siegel RL, Miller KD, Jemal A. Cancer statistics, 2015. CA Cancer J Clin. 2015; 65:5–29. 10.3322/caac.21254 25559415

[2] Zappa C, Mousa SA. Non-small cell lung cancer: current treatment and future advances. Transl Lung Cancer Res. 2016; 5:288–300. 10.21037/tlcr.2016.06.07 27413711 PMC4931124

[3] Higuchi T, Oshiro H, Zhang Z, Miyake K, Sugisawa N, Katsuya Y, Yamamoto N, Hayashi K, Kimura H, Miwa S, Igarashi K, Zhao M, Bouvet M, et al. Osimertinib regresses an EGFR-mutant cisplatinum- resistant lung adenocarcinoma growing in the brain in nude mice. Transl Oncol. 2019; 12:640–45. 10.1016/j.tranon.2019.01.007 30807997 PMC6393699

[4] Agirre X, Meydan C, Jiang Y, Garate L, Doane AS, Li Z, Verma A, Paiva B, Martín-Subero JI, Elemento O, Mason CE, Prosper F, Melnick A. Long non-coding RNAs discriminate the stages and gene regulatory states of human humoral immune response. Nat Commun. 2019; 10:821. 10.1038/s41467-019-08679-z 30778059 PMC6379396

[5] Li PF, Chen SC, Xia T, Jiang XM, Shao YF, Xiao BX, Guo JM. Non-coding RNAs and gastric cancer. World J Gastroenterol. 2014; 20:5411–19. 10.3748/wjg.v20.i18.5411 24833871 PMC4017056

[6] Lu CW, Zhou DD, Xie T, Hao JL, Pant OP, Lu CB, Liu XF. HOXA11 antisense long noncoding RNA (HOXA11-AS): a promising lncRNA in human cancers. Cancer Med. 2018; 7:3792–99. 10.1002/cam4.1571 29992790 PMC6089141

[7] Li S, Li J, Chen C, Zhang R, Wang K. Pan-cancer analysis of long non-coding RNA NEAT1 in various cancers. Genes Dis. 2017; 5:27–35. 10.1016/j.gendis.2017.11.003 30258932 PMC6146416

[8] Tomczak K, Czerwińska P, Wiznerowicz M. The cancer genome atlas (TCGA): an immeasurable source of knowledge. Contemp Oncol (Pozn). 2015; 19:A68–77. 10.5114/wo.2014.47136 25691825 PMC4322527

[9] Wu W, Liu F, Wu K, Chen Y, Wu H, Dai G, Zhang W. Lon peptidase 2, peroxisomal (LONP2) contributes to cervical carcinogenesis via oxidative stress. Med Sci Monit. 2018; 24:1310–20. 10.12659/msm.908966 29502128 PMC5846714

[10] Li J, Han L, Roebuck P, Diao L, Liu L, Yuan Y, Weinstein JN, Liang H. TANRIC: an interactive open platform to explore the function of lncRNAs in cancer. Cancer Res. 2015; 75:3728–37. 10.1158/0008-5472.CAN-15-0273 26208906 PMC4573884

[11] Li X, Wu X. MiR-21-5p promotes the progression of non-small-cell lung cancer by regulating the expression of SMAD7. Onco Targets Ther. 2018; 11:8445–54. 10.2147/OTT.S172393 30568467 PMC6276624

[12] Yan L, Ma J, Wang Y, Zan J, Wang Z, Zhu Y, Zhu Y, Ling L, Cao L, Liu X, Li S, Xu L, Qi Z, et al. miR-21-5p induces cell proliferation by targeting TGFBI in non-small cell lung cancer cells. Exp Ther Med. 2018; 16:4655–63. 10.3892/etm.2018.6752 30542417 PMC6257667

[13] Oka H, Kojima T, Ihara K, Kobayashi T, Nakano H. Comprehensive investigation of the gene expression system regulated by an aspergillus oryzae transcription factor XlnR using integrated mining of gSELEX-seq and microarray data. BMC Genomics. 2019; 20:16. 10.1186/s12864-018-5375-5 30621576 PMC6323846

[14] Chen WJ, Tang RX, He RQ, Li DY, Liang L, Zeng JH, Hu XH, Ma J, Li SK, Chen G. Clinical roles of the aberrantly expressed lncRNAs in lung squamous cell carcinoma: a study based on RNA-sequencing and microarray data mining. Oncotarget. 2017; 8:61282–304. 10.18632/oncotarget.18058 28977863 PMC5617423

[15] Li DY, Chen WJ, Shang J, Chen G, Li SK. Regulatory interactions between long noncoding RNA LINC00968 and miR-9-3p in non-small cell lung cancer: a bioinformatic analysis based on miRNA microarray, GEO and TCGA. Oncol Lett. 2018; 15:9487–97. 10.3892/ol.2018.8476 29805671 PMC5958761

[16] Xiu DH, Liu GF, Yu SN, Li LY, Zhao GQ, Liu L, Li XF. Long non-coding RNA LINC00968 attenuates drug resistance of breast cancer cells through inhibiting the Wnt2/β-catenin signaling pathway by regulating Wnt2. J Exp Clin Cancer Res. 2019; 38:94. 10.1186/s13046-019-1100-8 30791958 PMC6385430

[17] Fujii M, Toyoda T, Nakanishi H, Yatabe Y, Sato A, Matsudaira Y, Ito H, Murakami H, Kondo Y, Kondo E, Hida T, Tsujimura T, Osada H, Sekido Y. TGF-β synergizes with defects in the hippo pathway to stimulate human Malignant mesothelioma growth. J Exp Med. 2012; 209:479–94. 10.1084/jem.20111653 22329991 PMC3302232

[18] Yu Y, Gu S, Li W, Sun C, Chen F, Xiao M, Wang L, Xu D, Li Y, Ding C, Xia Z, Li Y, Ye S, et al. Smad7 enables STAT3 activation and promotes pluripotency independent of TGF-β signaling. Proc Natl Acad Sci USA. 2017; 114:10113–18. 10.1073/pnas.1705755114 28874583 PMC5617276

[19] Gao W, Su R, Sun W, Wang QZ, Lv XY, Bao JJ, Yu JR, Wang LH, Musa HH, Chen L. Temporal and spatial expression of smads and their correlation with YAP1 expression in sheep. Genet Mol Res. 2016; 15. 10.4238/gmr.15037715 27706608

[20] Franco-Zorrilla JM, Valli A, Todesco M, Mateos I, Puga MI, Rubio-Somoza I, Leyva A, Weigel D, García JA, Paz-Ares J. Target mimicry provides a new mechanism for regulation of microRNA activity. Nat Genet. 2007; 39:1033–37. 10.1038/ng2079 17643101

[21] Tay Y, Rinn J, Pandolfi PP. The multilayered complexity of ceRNA crosstalk and competition. Nature. 2014; 505:344–52. 10.1038/nature12986 24429633 PMC4113481

[22] Ren W, Hou J, Yang C, Wang H, Wu S, Wu Y, Zhao X, Lu C. Extracellular vesicles secreted by hypoxia pre-challenged mesenchymal stem cells promote non-small cell lung cancer cell growth and mobility as well as macrophage M2 polarization via miR-21-5p delivery. J Exp Clin Cancer Res. 2019; 38:62. 10.1186/s13046-019-1027-0 30736829 PMC6367822

[23] Győrffy B, Surowiak P, Budczies J, Lánczky A. Online survival analysis software to assess the prognostic value of biomarkers using transcriptomic data in non-small-cell lung cancer. PLoS One. 2013; 8:e82241. 10.1371/journal.pone.0082241 24367507 PMC3867325

[24] Tong Y, Ru B, Zhang J. miRNACancerMAP: an integrative web server inferring miRNA regulation network for cancer. Bioinformatics. 2018; 34:3211–13. 10.1093/bioinformatics/bty320 29897412

[25] Uhlén M, Fagerberg L, Hallström BM, Lindskog C, Oksvold P, Mardinoglu A, Sivertsson Å, Kampf C, Sjöstedt E, Asplund A, Olsson I, Edlund K, Lundberg E, et al. Proteomics. Tissue-based map of the human proteome. Science. 2015; 347:1260419. 10.1126/science.1260419 25613900

