# Supplementary material for: LINC00968 can inhibit the progression of lung adenocarcinoma through the miR-21-5p/SMAD7 signal axis
Source: Aging (Albany NY). 2020 Nov 4;12(21):21904–22. doi: 10.18632/aging.104011 (PMC7695398; doi:10.18632/aging.104011)
Supplement: Supplementary Figures [file aging-12-104011-s001..pdf]

SUPPLEMENTARY FIGURES

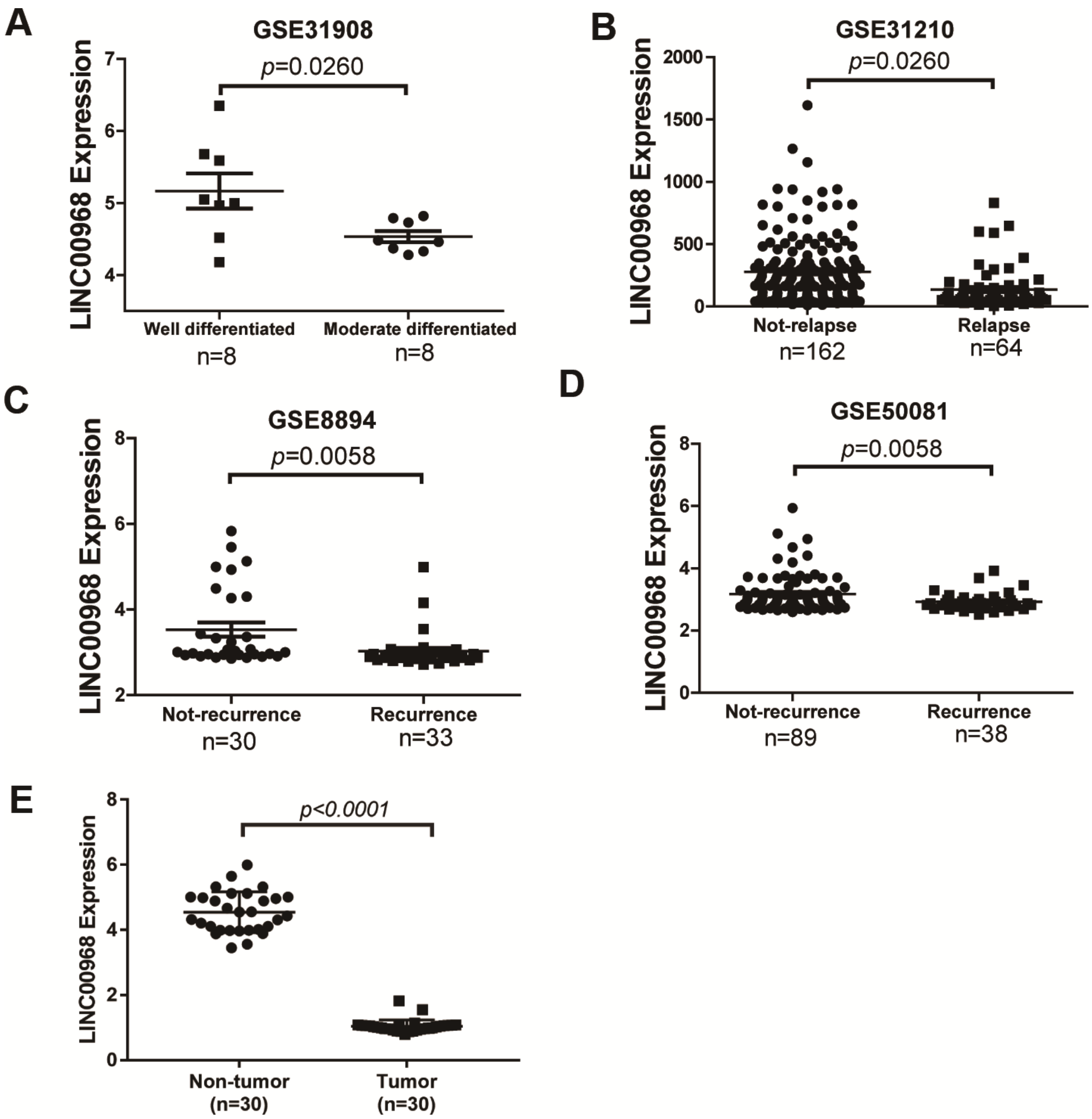

Supplementary Figure 1. (**A**) The expression of LINC00968 was lower in the moderate differentiation tissues than in well differentiation tissues based on GSE31908 dataset. The patients with relapse (**B**) had low LINC00968 expression as compared to those without relapse. The expression of LINC00968 was decreased in patients with recurrence compared to patients without recurrence based on GSE8894 (**C**) and GSE50081 (**D**). (**E**) RT-PCR detection of the expression of LINC00968 in 30 cases of LUAD tissues and adjacent tissues.

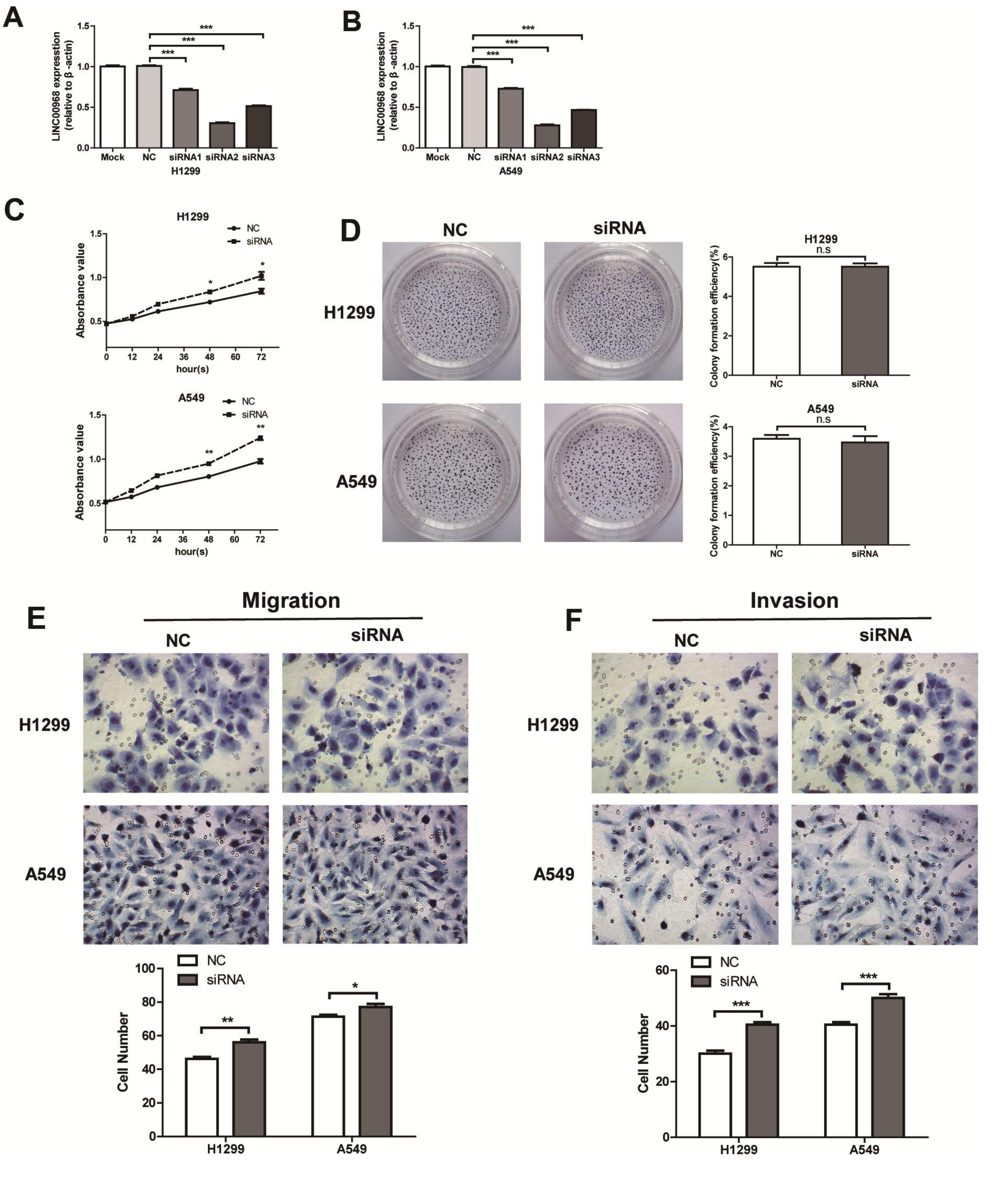

Supplementary Figure 2. LINC00968 knockdown promotes malignant phenotype of tumor cell. The transfection efficiency of LINC00968 siRNAs was successful in H1299 (**A**) and A549 (**B**) cells as detected by fluorescence microscope and qRT-PCR. The second LINC00968 siRNA (siRNA2) showed the best inhibitory effect of LINC00968 expression, which was used for the following experiments. (**C**) Cell proliferation was increased after LINC00968 knockdown at 48 and 72 h. (**D**) However, the colony forming ability did not differ significantly between LINC00968 knockdown cells and control cells. The migration (**E**) and invasion (**F**) ability of LINC00968-deficiency cells was enhanced compared to the control cells. *$P<0.05$, **$P<0.01$, ***$P<0.001$.

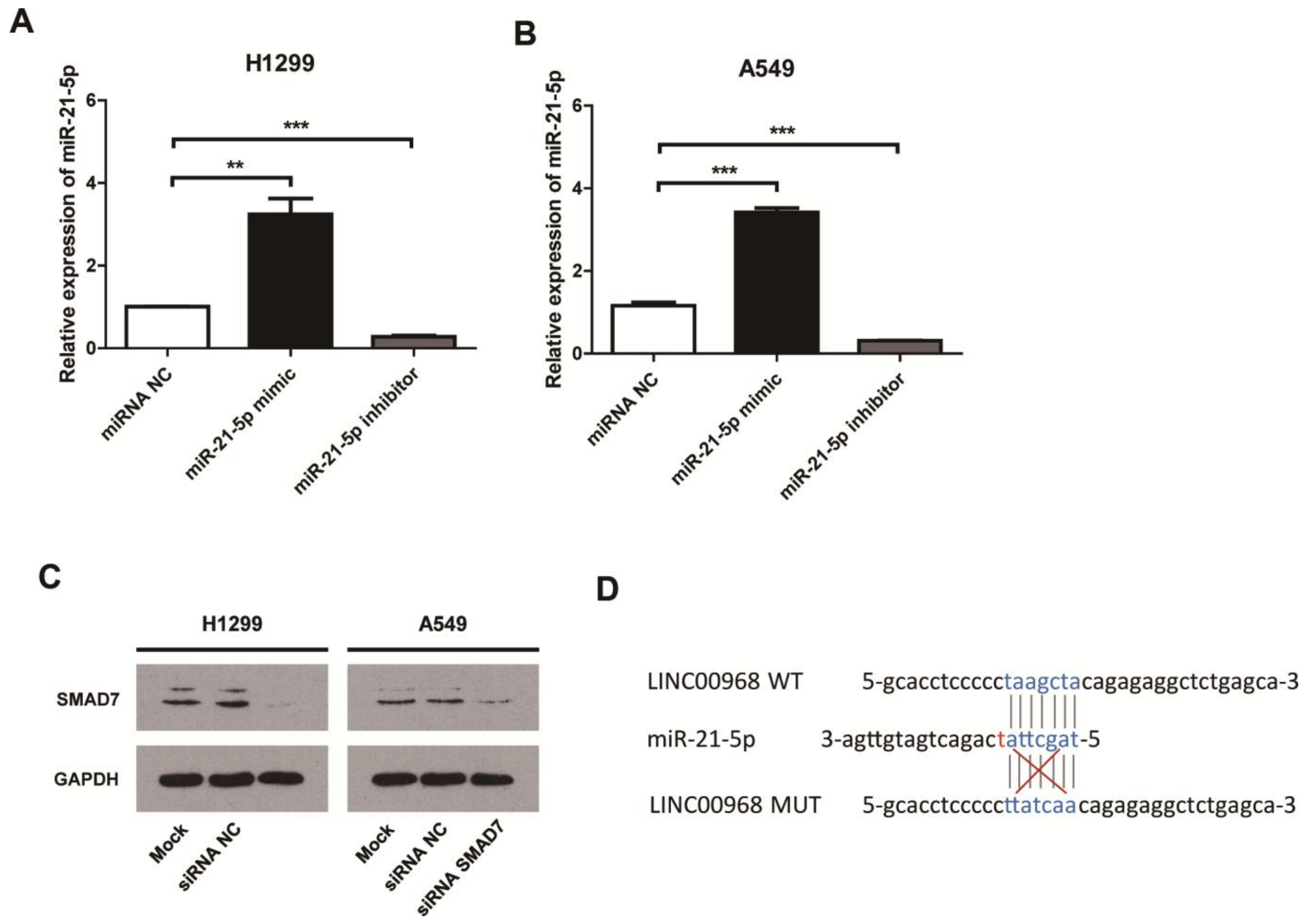

Supplementary Figure 3. miR-21-5p mimics and inhibitor were transfected into H1299 (**A**) and A549 (**B**) cells, respectively. The levels of miR-21-5p expression were validated by qRT-PCR. (**C**) The SMAD7 targeting siRNA were used to silence the expression of SMAD7. The knock down effect was detected by western blotting. (**D**) The mutation sequence of LINC00968 **P<0.01, ***P<0.001.

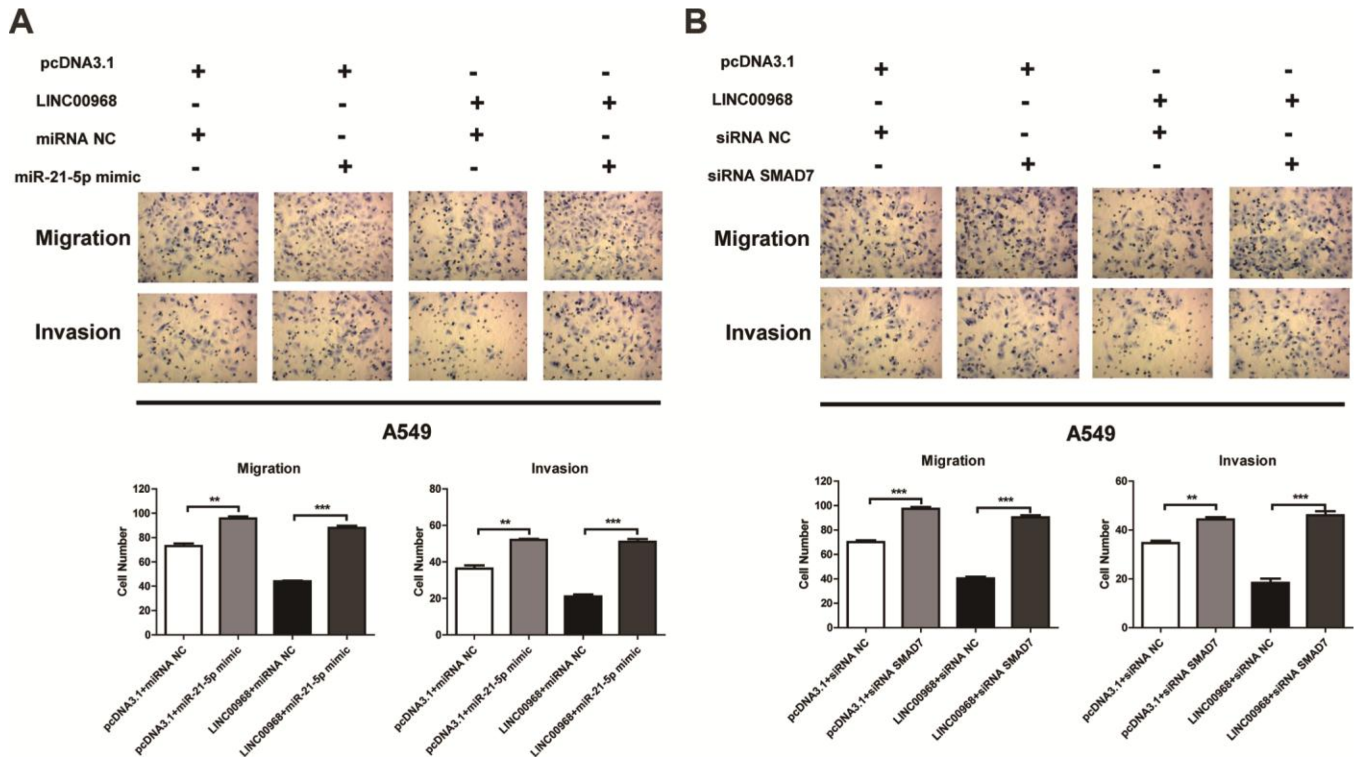

Supplementary Figure 4. The function of LINC00968/miR-21-5p/SMAD7 axis on migration and invasion of A549 cells. (**A**) Overexpression of LINC00968 inhibited tumor cell migration and invasion, whereas upregulation of miR-21-5p partially attenuated the inhibitory effects of LINC00968 over-expression. (**B**) The tumor-promoting actions of SMAD7 knockdown on cell migration and invasion were partially reversed by LINC00968 overexpression. ***P*<0.01, ****P*<0.001.
